# P250: Nosocmial infections at the hospital principal of Dakar (Senegal): assessment surveys "1 given day" from 2006 to 2010

**DOI:** 10.1186/2047-2994-2-S1-P250

**Published:** 2013-06-20

**Authors:** B Fall, L Nadiel, M Ndiaye, MN Seye, E Diémé, KS Ndiaye, S Diawara, Y Diémé, B Wade

**Affiliations:** 1 Laboratories federtaion, Dakar, Senegal; 2 Direction, HPD, Dakar, Senegal; 3 Reanimation service, HPD, Dakar Senegal; 4 Pediatrics, HPD, Dakar, Senegal; 5 Surgical Department, HPD, Dakar, Senegal

## Introduction

The lack of surveillance data on nosocomial infections is one of the deficiencies of health systems in Africa. Hospital Principal of Dakar intends to be an exception in this regard.

## Objectives

Our objective was to investigate the epidemiological, clinical and bacteriological characteristics of these infections in order to reach a better orientation of prevention activities.

## Methods

Based on a standardized protocol recommended by the national program (PRONALIN) a group of internal, trained investigators of the hospital is deployed in the different services on one specific day from 2006 to 2010. This happens concomitantly in all hospitals in the country. All patients occupying a hospital bed the day of the survey were concerned and any infection occurring after 48 hours of hospitalization was considered nosocomial. The data are validated by a pool of biologists and subsequently analyzed by Epi-Info version 2003.

## Results

One thousand and three hundred ninety seven (1397) patients were included. The overall prevalence was 4.24% (respectively 6.9, 4.8, 3.7, 3.6 and 2.6% from 2006 to 2010). The average age was 37 years [1, 108 years] and the sex-ratio was 1.15. The most affected services were respectively pediatrics (29.4%), surgery (25%), medicine (22%) and intensive care units (20.6%). Bacteremias were the most common infections (33.3%) followed by urinary tract infections (24.3%), surgical site infections (15.2%) and lung infections (15.1%). The other sites accounted for 15.1% of cases. The microorganisms most commonly responsible for nosocomial infections were: *Enterobacter* (21.4%), *Klebsiella* (20%) and *Pseudomonas aeruginosa* (18.6%). ESBL-producing enterobacteria were implicated in 34% of cases and those producing derepressed cephalosporinase in 12.8%.

## Conclusion

This study shows the important place occupied by multi-resistant bacteria and illustrates the efforts that are still required in some services, despite a general trend towards lower rates of nosocomial infections in recent years.

## Competing interests

None declared

